# ID 361 - Transferência de Tecnologia em Saúde em Projetos de Cooperação Bilateral entre o Brasil e os Países da América Latina e Caribe, 2008-2023

**DOI:** 10.5327/2237-9622.2025.v34s1.361

**Published:** 2025-11-25

**Authors:** Thaís Barbosa de Oliveira, Thaliane Barbosa de Oliveira, Rafaela de Paula Sales, Aline Gonçalves Pereira

**Keywords:** tecnologia em saúde, cooperação técnica entre países, transferência de tecnologia

## Abstract

**Introdução:**

A cooperação bilateral em saúde configura-se como uma importante estratégia de diplomacia internacional para fortalecimento dos sistemas de saúde. No contexto da América Latina e do Caribe (ALC), têm-se cenários regionais e geográficos comuns, o que corrobora para uma articulação diplomática em torno de inovações e transferência de tecnologias em saúde. O Brasil, historicamente, assume o protagonismo no compartilhamento de tecnologias em saúde com a ALC. Diante disso, este estudo objetivou descrever os projetos de cooperação bilateral entre o Brasil e a ALC que pautaram a transferência de tecnologias em saúde.

**Método:**

Os dados foram extraídos da plataforma pública da Associação Brasileira de Cooperação (ABC) do Ministério das Relações Exteriores. Foram levantadas informações de projetos de cooperação internacional de países da ALC entre os anos de 2008 e 2023, com classificação em planilha Excel. Os objetivos dos projetos foram analisados e classificados em grandes temáticas comuns.

**Resultados:**

Identificou-se o total de 45 projetos de cooperação bilateral, entre o Brasil e 14 países da ALC, que pautaram a transferência de tecnologias em saúde. O ano de 2009 (37,78%, n=17) apresentou o maior número de projetos iniciados quando em comparação aos demais anos. Entre os 14 países identificados nas cooperações, 7 (50,00%) são da América do Sul, 3 (21,14%) da América Central, 3 (21,14%) do Caribe e 1 (7,14%) da América do Norte. Quando se observa o total de projetos por país, Bolívia (n=4) e Equador (n=4) apresentaram a maior quantidade de projetos na América do Sul; Cuba (n=5) no Caribe; e Panamá (n=4) na América Central. Diante disso, houve predominância de projetos na América do Sul (55,56%, n=25), seguida pelo Caribe (n=08) e pela América Central (17,78%, n=7). As principais temáticas dos projetos foram direcionadas à transferência de tecnologias e conhecimentos para: (A) a vigilância em saúde ambiental e o enfrentamento de doenças transmissíveis (35,56%, n=16); e (B) a instituição de bancos de leite humano (33,33%, n=15). Nos projetos do grupo A, as principais tecnologias transferidas trataram de técnicas epidemiológicas de análise de dados e controle de vetores. Para o grupo B, trataram de tecnologias de processamento e controle da qualidade de leite humano.

**Conclusão:**

O ano de 2009 predominou com a maior quantidade de projetos, indicando que o País apresentou uma política externa mais ativa, buscando ampliar sua presença internacional. As principais temáticas trabalhadas vão ao encontro das necessidades dos países no enfrentamento de doenças transmissíveis e prevenção da mortalidade materno-infantil. A predominância de projetos na América do Sul pode ser atribuída à proximidade geográfica e às semelhanças culturais e epidemiológicas entre os países. Bolívia, Equador, Cuba e Panamá se destacam como os países com maior número de projetos, o que pode refletir uma maior necessidade ou capacidade de absorção de tecnologias de saúde, bem como uma relação mais estreita com o Brasil em termos de cooperação técnica. Entre as limitações deste estudo, destaca-se que a plataforma da ABC pode não estar completamento atualizada. Ademais, não foram levantados termos ou acordos de cooperação bilateral, uma vez que os objetivos deste estudo se limitaram ao levantamento de projetos datados pela ABC. Apesar disso, as hipóteses aqui levantadas podem subsidiar estudos futuros.

